# An Experimental Study on the Action of Pilocarpin

**Published:** 1883-05

**Authors:** Julius Pohlman

**Affiliations:** Buffalo, N. Y.


### An Experimental Study on the Action of Pilocarpin.

By Dr. Julius Pohlman, Buffalo, N. Y.

In the proceedings of the American Association for the Advancement of Science, at the Cincinnati meeting, 1881, I find the record of an observation made by Dr. D. W. Prentiss, of Washington, D. C., on the changes in the color of human hair produced by the administration of pilocarpin.

In order to obtain a little more accurate knowledge upon this subject, I determined to test by experiments, and to that end procured, on September 17, 1882, two Albino rabbits, one of which was treated, while the other was kept on the same food and under the same conditions for reference, to enable me to detect any change going on in the coloring of the hair.

The experiment consisted in the daily administration of three hypodermic injections of pilocarpin, aggregating to 1, 1¼ and 1½ grains, dissolved in half an ounce of water, per week. As I did not intend to observe the diaphoretic effects of the drug, and only wanted to establish its action upon the color of the hair, I did not increase the dose to any extent.

The rabbit received from

| | | | | | |
|---|---|---|---|---|---|
| September | 18th to | September | 25th, | 1 | grain. |
| " | 25th to | October | 2d, | 1 | " |
| October | 2d to | " | 9th, | 1¼ | " |
| " | 9th to | " | 16th, | 1¼ | " |
| " | 16th to | " | 23d, | 1½ | " |

I discontinued this experiment on October 23d; the result was entirely negative. There was not the slightest change in the coloring of the hair and eyes; the fur was as white and the eyes were as red on October 23d as on September 18th. During all this time the health of the animal did not seem to suffer any; there was no salivation; appetite and strength, the latter well attested by its powerful kicking, never failed; it kept itself neat and clean, and did not seem to suffer any inconvenience outside of the pricking of the needle of the hypodermic syringe.

I am sorry that I did not make a post mortem examination of this rabbit at the time I discontinued the experiment.

After the Albino rabbit had been under treatment two weeks, I thought that the drug might, perhaps, act better upon lightly-colored animals, and for this reason procured two other rabbits, male and female, of the same size and age. The male, *A*, on which I experimented, had the following markings: A patch of rusty brownish-blackish color between eye and ear on each side of the head extended anteriorly in a narrow streak around the eye, and posteriorly to the base of the ear, which had a blackish color. The rest of the body of the animal was white, with the exception of three small narrow patches of a light rusty-yellow color along the spinal column, and one on the tail.

The colored female rabbit, *B*, was of a uniform light brown, and kept on the same food and under the same conditions as my patient. Samples of hair were clipped from it at the beginning and at the end of the experiment to meet any objection that would ascribe the darkening of the hair to increased age.

*A* was treated to the following quantities of pilocarpin, administered in hypodermic injections three times daily:

From October 2d to October 9th, 1 grain.
" " 9th to " 16th, 1 "

At the end of this week the color on the back had darkened a little.

From October 16th to October 23d, 1 grain.

During the course of this week a faint spreading of the color over the posterior half of the animal was perceptible along the spinal column towards the tail and the upper parts of the hind legs.

From October 23d to October 30th, 1½ grain.

The color kept spreading more, slowly appearing in faint small patches on the sides of the animal. The original color on its back had almost reached its maximum depth at the end of the week and consisted of a chocolate brown. The intensity of the induced coloring kept increasing from the spinal column downward.

From October 30th to November 6th, 1½ grain.

The dose seemed too large, judging from the quiet and drooping attitude of the animal towards the latter end of this week, and I therefore resorted again to the smaller original doses.

| | | | | | |
|---|---|---|---|---|---|
| From | November | 6th to | November | 13th, | 1 grain. |
| " | " | 13th to | " | 20th, | 1 " |
| " | " | 20th to | " | 27th, | 1 " |

The colored part of the animal had increased constantly in size, and toward the end of the month covered almost the whole posterior half of the animal's body, including hind legs and tail; faint patches began to appear a little back of the shoulders.

| | | | | | |
|---|---|---|---|---|---|
| From | November | 27th to | December | 4th, | 1 grain. |
| " | December | 4th to | " | 11th, | 1 " |
| " | " | 11th to | " | 16th, | 1 " |

The induced coloring of the animal had deepened in tone considerably and covered about the posterior two-thirds of its body. The peculiarity of it consists in the fact, that when the hair is lifted by a motion of the hand from behind forward, the animal appears darker. The coloring matter has gradually forced its way outward along the hair, and large numbers of their tips are still white where the pigment has not yet had sufficient time to make its way through the whole length of the hair. In contradistinction to this, the natural color of the hair of animals is, as a rule, darkest at or near the tips.

To watch the slow increase of the color, both in quantity and in quality, was a matter of considerable interest. Small patches, in their infancy of coloring, could be detected by a lifting of the hair when the smooth fur looked as yet pure white. Slowly and gradually the first faint color would darken and extend outward, from week to week, and I am sorry to be at present unable to say how far this artificial coloring process could have been extended.

There was no increase in size or depth of color in any of the dark portions of the head, nor did the eyes darken any. All the increase of pigment was confined to the posterior portions of the body.

With the exception of a few days at the beginning of November, the animal apparently enjoyed good health, and did not evince any signs of inconvenience or sickness after receiving its daily ration of hypodermic injection in the morning, at noon and in the evening.

Rabbit *B* did not change the color of its hair the slightest, as was testified by a comparison of the samples of hair taken at the beginning and at the end of the above-described experiment.

It would be interesting to find out whether a pair of animals, colored artificially in this way, would transmit the original or the induced color to their offsprings; also, whether a disease, induced by the continued use of pilocarpin, would be hereditary; and whether the artificial colors would be permanent or again disappear with the discontinuance of the use of the drug. There is likewise a possibility that the medicine may act more powerfully on carnivorous than on herbivorous animals, and my future experiments in this line will include dogs.

On December 17, 1882, the two rabbits were killed for the purpose of a post mortem examination. Heart, lungs, liver, stomach and kidneys were in a normal condition in both animals, but spleen and supra-renal capsules presented a striking difference. The spleen of *A* was enlarged to about twice its natural size and crossed in various directions by strong bands of connective tissue. The supra-renal capsules of *B*, of a pale, yellow color outside, presented on longitudinal section the cortical and medullary substance sharply defined. The former of the same pale yellow, the latter of a fleshy color, darkest at its exterior margin. The capsules of *A* were a little larger than those of *B*, and of the same appearance outside. A longitudinal section revealed a very ill-defined division between the two substances; the distinct reddish color of the medullary portion in the healthy animal had almost entirely disappeared, and only a faint streak along the center of the section indicated its presence. The cut surface of the cortical substance had a reddish color which gradually merged into the small semi-translucent fleshy colored mass of the medullary substance. A microscopic examination bears testimony of this destructive process in the capsules. As my preparations were not stained, I am unable to record the nature of the changes. The best view of the sections I obtained by polarized light with a $\frac{4}{10}$ Beck's objective and low power eye-piece. The sections were kindly made for me by Dr. Geo. E. Fell, of this city.

Of course, it would be premature to theorize or draw any deductions from these single and incomplete experiments. I think, however, that I have succeeded in demonstrating the capability of pilocarpin to darken the color of the hair in the presence of pigmentary matter, perhaps by a stimulating action on its formation; but that the drug is unable to produce color where pigmentary matter is absent, as shown in the case of the Albino rabbits.

Whether the pathological condition of the supra-renal capsules and the spleen had any connection with the increased formation of pigment (vide Addison's disease) or was only an incidental effect of the drug, I hope to demonstrate more fully during the year.

The rabbit on which the above-described experiments were made is at present in the museum of the University of Buffalo.

